# High human papillomavirus prevalence among females attending high school in the Eastern Cape Province of South Africa

**DOI:** 10.1371/journal.pone.0253074

**Published:** 2021-06-18

**Authors:** Zizipho Z. A. Mbulawa, Nontuthuzelo I. Somdyala, Sikhumbuzo A. Mabunda, Anna-Lise Williamson

**Affiliations:** 1 National Health Laboratory Service, Nelson Mandela Academic Hospital, Mthatha, South Africa; 2 Department of Laboratory Medicine and Pathology, Walter Sisulu University, Mthatha, South Africa; 3 SAMRC/UCT Gynaecological Cancer Research Centre, University of Cape Town, Cape Town, South Africa; 4 Division of Medical Virology, Department of Pathology, University of Cape Town, Cape Town, South Africa; 5 South African Medical Research Council, Burden of Disease Research Unit, Cape Town, South Africa; 6 The George Institute for Global Health, University of New South Wales, Sydney, Australia; 7 Institute of Infectious Disease and Molecular Medicine, University of Cape Town, Cape Town, South Africa; University of Nebraska-Lincoln, UNITED STATES

## Abstract

As part of the human papillomavirus (HPV) vaccination strategy in South Africa, it is essential to have information on HPV prevalence, and HPV types distribution among the unvaccinated population. Information on the prevalence of HPV and the distribution of HPV types in adolescents and young women in South Africa’s Eastern Cape Province is minimal. Therefore, this study investigates the prevalence, distribution of HPV types, and factors associated with HPV infection amongst unvaccinated female learners. A sample composed of 213 sexually active female learners attending high schools in the Eastern Cape Province of South Africa; median age 18 years, who provided self-collected vaginal specimens. Roche Linear Array HPV genotyping assay that detects 37 HPV genotypes was used to detect HPV infection. HPV infection was detected in 76.06% (162/213) of participants. Of these 14.55% (31/213) were positive for HPV types targeted by the Cervarix® HPV vaccine (HPV-16 and/or 18), 20.66% (44/213) by Gardasil®4 (HPV-6, -11, -16 and/or -18) and 37.09% (79/213) by Gardasil®9 (HPV-6, -11, -16, -18, -31, -33, -45, -52 and/or -58). HPV-35, commonly detected in cervical cancer cases among women of African ancestry, was frequently detected (9.40%). Participants who reported to have ever consumed alcohol had a significantly higher risk of HPV infection (OR: 2.91, 95% CI: 1.38–6.11, p = 0.005). High HPV prevalence was observed among participants. The high prevalence of HPV types targeted by the Gardasil®9 vaccine encourages the introduction of the Gardasil®9 vaccine. Data from this study will inform both vaccination campaigns and monitor the impact on HPV types after vaccination.

## Introduction

Sexually transmitted infection (STI) prevalence is very high in developing countries where routine STI screening and treatment have inadequate coverage. Self-sampling for STIs, including human papillomavirus (HPV) testing, is a promising method to increase the STI screening coverage [1,2]. Self-collected specimen for detecting HPV has been widely used in women and is highly acceptable by women, including adolescents from both rural and urban areas [3,4]. A good-agreement of HPV prevalence between self-collected and clinician-collected samples has been reported worldwide [5,6], including among South African adolescents (16–17 years) [7].

HPV is the most common sexually transmitted virus [8–11], with its peak prevalence observed in adolescents and young women soon after sexual debut and decreasing with increasing age in women [12,13]. HPV prevalence (low and high-risk) ranges between 44% and 85% among South African adolescents and young women (15–25 years) [14–20]. Both cervical cancer and human immunodeficiency virus (HIV) burden is high in Africa [21–23]. Compared to HIV-negative individuals, HIV-infected individuals are more likely to be infected by HPV, co-infected with multiple HPV types, persistent infection, reactivation, and develop HPV-associated cancers on different anatomical sites [23–26].

Cervical cancer is the second most common cancer in South African women. The National Cancer Registry (NCR) of South Africa reported an age-standardized rate (ASR) of 22.56 per 100,000 for all South African women in 2014 and ASR of 27.01 per 100,000 for black African women [27]. According to Somdyala et al. in the rural Eastern Cape Province of South Africa, the cervical cancer annual ASR per 100,000 increased from 22.0 in 1998–2002 to 29.2 in 2008–2012 [28]. Unfortunately, in South Africa, cervical cancer screening coverage is very low [29]. Makura and colleagues reported that between 2013 and 2014, less than 50% of the Eastern Cape Province women aged 30 years received Pap smear.

Currently, there are three HPV vaccines approved by the U.S Food and Drug Administration. They are Cervarix® (GlaxoSmithKline), Gardasil®4 (Merck Inc), and Gardasil®9 (Merck Inc). They target a different combination of HPV types; Cervarix® targets HPV-16/18, the most carcinogenic HPV types associated with approximately 70% cervical cancer cases; Gardasil®4 targets HPV-16/18 as well as two low-risk (LR) types, HPV-6/11, associated with genital warts and recurrent respiratory papillomatosis; and Gardasil®9 (Merck Inc) targets same types as Gardasil®4 and the other five high-risk (HR) HPV types HPV-31/33/45/52/58 [22,30–32]. Among HPV types known to be dominant in cervical cancer cases worldwide, HPV-16 is the most common type worldwide. It is important to note that among women of African ancestry origin, HPV-35 is detected in approximately 10% of cervical cancer cases, while it is detected in approximately 2% of worldwide cases [33–35]. The current HPV vaccines do not target HPV-35; the addition of HPV-35 to the Gardasil®9 types would increase the protection against HPV associated diseases among women of African ancestry [34].

Many countries have implemented HPV vaccination programs since the first licensure of Gardasil®4 in 2006 [31,32]. In South Africa, the school-based national HPV vaccination program was introduced in 2014, targeting girls aged nine years or older (mostly in grade-4), and the Cervarix® HPV vaccine two-dose schedule is used in this program. The vaccine schedules are 6-months apart within the academic calendar year [30,36,37]. As part of the HPV vaccination strategy in South Africa, it is essential to have information on HPV prevalence and HPV types distribution among the unvaccinated population to both inform vaccination campaigns as well as to monitor the impact on HPV types after vaccination [38]. Information on the prevalence of HPV and the distribution of HPV types in adolescents and young women in South Africa’s Eastern Cape Province is minimal. Therefore, this study aimed to investigate the prevalence and distribution of HPV genotypes; and factors associated with HPV amongst high school female learners in Eastern Cape, South Africa.

## Materials and methods

### Ethics statement

This study was approved by the University of Cape Town Human Research Ethics Committee (HREC: 369/2015). Permission to conduct research in the Eastern Cape was granted by the Eastern Cape Provincial Health Research Committee (EC_2016RP29_562). Both the Provincial Department of Health and Education in the Eastern Cape granted permission to investigators to conduct this study. Participation in the study was voluntary, with written informed consent and parental assent obtained for participants younger than 18 years.

### Study population and specimen collection

Participants of this study were recruited from grade 8 to 12 learners who participated in the HPV education intervention study conducted between April and May 2019 in two high schools situated in Chris Hani District Municipality of the Eastern Cape Province, South Africa. The participating schools belong to quintile one (no-fee paying schools) South African Department of education quintile ranking. The high schools were randomly selected. After HPV education intervention, females (≥15 years, regardless of sexual history status) were invited to the nearest primary care facilities where the study was conducted. Participants received information on study procedures, objectives, and other important information. After which, they responded to closed-ended questionnaires enquiring about their demographics, sexual practices, contraceptive use, smoking habits, and alcohol consumption. Questionnaires were self-administered while the researcher was reading out questions and explaining where necessary. Pre and post-HIV counseling, rapid HIV tests were conducted by a qualified clinic staff member or HIV lay counselor. All participants with a positive HIV test had necessary follow-up according to the Health Department’s protocol guidelines.

The study reports on results only for those participants who were sexually experienced. A total of 257 female learners responded to the invitation, of whom 221 were sexually experienced, while 36 were not (Table 1). Self-collected vaginal specimens were only obtained from sexually experienced females who were not menstruating on the day of the visit (as per self-report). Only one participant noticed menstruation before collecting the specimen and was, therefore, excluded. A total of 220 sexually experienced participants provided self-collected vaginal specimens. A health professional demonstrated to study participants on how to collect specimen using the Evalyn® Brush (Rovers® Medical Devices B.V. Oss, Netherlands). Instruction leaflets that Rovers® Medical Devices provided were also used as a reference. In order to collect the self-collected vaginal specimen, participants were requested to gently insert the Evalyn® Brush as far as possible into the vagina while in a standing or squatting position, rotate the brush five times in the same direction, remove the brush, pull back the plunger till the brush enters the casing and place the cap back on the Evalyn® Brush. Participants were assured that the demonstration brush used for specimen collection was soft and allowed to touch it. The Evalyn® Brushes with vaginal specimens were stored at room temperature and transported to the University of Cape Town HPV laboratory within 30 days.

**Table 1:** Brief demographic characteristics of high school learners who visited the clinic.

Characteristics	Female (N = 257)	
Age, years; median (IQR*)	18	(17–19)
**Age, years; n (%)**		
**15–16**	41	(15.95)
**17–19**	159	(61.87)
**20–22**	57	(22.18)
**Grade; n (%)**		
**Grade 8**	19	(7.48)
**Grade 9**	29	(11.42)
**Grade 10**	68	(26.77)
**Grade 11**	73	(28.74)
**Grade 12**	65	(25.59)
**Not stated**	3	(1.17)
**Sexually active; n (%)**		
**Yes**	221	(85.99)
**No**	36	(14.01)
**HIV status; n (%)**		
**Positive**	9	(3.50)
**Negative**	172	(66.93)
**Not tested**	76	(29.57)
**Ever smoked; n (%)**		
**Yes**	39	(15.18)
**No**	218	(84.82)
**Age started drinking alcohol (Years); n (%)**		
**≤12**	15	(5.84)
**13–14**	39	(15.18)
**15–16**	84	(32.68)
**≥17**	65	(25.29)
**Don’t recall**	54	(21.01)

*IQR = Interquartile Range = 25^th^ percentile and 75^th^ percentile.

### Nucleic acid extraction

The white brush part was detached from the Evalyn® Brush device and placed in sterile 2ml cryo-tubes. One milliliter of Digene specimen transport medium (Qiagen, Hilden, Germany) was added and vortexed three times, 10 minutes apart, to detach the cells from the white brush. A total of 400μl were used for nucleic acid extraction that was conducted using an automated procedure of MagNA Pure Compact (Roche Molecular Systems, Inc., Branchburg, NJ, USA) and MagNA Pure Compact Nucleic Acid Isolation Kit (Roche Molecular Systems, Inc., Branchburg, NJ, USA).

### HPV detection

Roche Linear Array HPV Genotyping Test (Roche Molecular Systems, Inc., Branchburg, NJ, USA) was used to detect HPV genotypes in extracted nucleic acid from vaginal specimens and manufacturer instructions were followed. The Linear Array HPV Genotyping Test amplifies the target HPV DNA for 37 anogenital HPV genotypes and include 24 low risk (LR) HPV types (HPV-6, -11, -26, -40, -42, -53, -54, -55, -61, -62, -64, -66, -67, -69, -70, -71, -72, -73, -81, -82, -83, -84, -IS39 and -CP6108) and 13 high risk (HR)-HPV types (HPV-16, -18, -31, -33, -35, -39, -45, -51, -52, -56, -58, -59 and -68). The Roche Linear Array HPV Genotyping Test also amplifies β-globin gene to monitor sample adequacy, extraction, amplification and hybridization.

### Data analysis

All variables were captured and coded in Microsoft excel 2013 and exported to Stata 14.1 for analysis. Participants were counted more than once when determining the prevalence of LR-HPV, HR-HPV, and probable HR-HPV if they have types that belong to more than one category. Multiple HPV infections were defined as the detection of two or more HPV types in the same sample. Numerical variables were explored using the Shapiro Wilk test, histogram, and/or box-and-whisker plot. The median and interquartile range (IQR) are used to summarise age in years since they were not normally distributed. Age was later categorised into three categories. The Wilcoxon rank-sum test was used to compare the median ages of males and females. Categorical variables are presented using frequency tables, percentages, and graphs. The two-sample test of proportions was performed to compare demographic characteristics of males and females, HPV status by the study site, and the age gap in years of current and/or previous sexual partner(s). Logistics regression was used to determine the bivariate association of HPV and the other variables to determine the Odds Ratio (OR). The unadjusted model (bivariate associations) is presented. The 95% Confidence Interval (CI) was used to estimate the precision of estimates. The level of significance was set at 5% (p-value ≤ 0.05) for statistical significance.

## Results

### Demographic characteristics of study participants

A total of 220 sexually experienced females provided self-collected vaginal specimens; however, 3.18% (7/220) were β-globin negative and excluded from the analysis. Participants with valid (positive β-globin) vaginal specimens had a median age of 18 years (IQR: 17–19 years). A proportion of 32.39% study participants reported a sexual debut of age 16 years, 53.99% had 2–3 current sexual partners, 35.68% used condoms during last sexual intercourse, 79.44% had vaginal sex in the past month, 20.66% had anal sex in the past month, 16.43% had oral sex in the past month, 62.91% were on contraceptives, 22.07% had been pregnant, 65.73% had experienced vaginal discharge, and 21.13% had experienced genital warts/blisters/ulcers (Table 2).

**Table 2:** The demographic characteristics of sexually active high school female learners who participated in the HPV prevalence study (N = 213).

Variable		n	(%)
**Age (years)**	15–16	19	(8.92)
	17–19	137	(64.32)
	20–22	57	(26.76)
**Grade**	Grade 8	12	(5.69)
	Grade 9	17	(8.06)
	Grade 10	55	(26.07)
	Grade 11	64	(30.33)
	Grade 12	63	(29.86)
	Not stated	2	(0.94)
**HIV**	Positive	8	(3.76)
	Negative	139	(65.26)
	Not tested	66	(30.99)
**Sexual debut (Years)**	≤13	12	(5.63)
	14	12	(5.63)
	15	60	(28.17)
	16	69	(32.39)
	≥17	59	(27.70)
	Not stated	1	(0.47)
**Current Sexual partners**	None	1	(0.47)
	1	49	(23.00)
	2–3	115	(53.99)
	4–6	34	(15.96)
	Not stated	14	(6.57)
**Number of Sexual partners in the past 3-months**	None	33	(15.49)
	1	141	(66.20)
	2–3	24	(11.27)
	4	1	(0.47)
	Not stated	14	(6.57)
**Ever consumed alcohol**	Yes	175	(82.16)
	No	38	(17.84)
**Drunk during last intercourse**	Yes	12	(5.63)
	No	187	(87.79)
	Not stated	14	(6.57)
**Condom used during last intercourse**	Yes	76	(35.68)
	No	123	(57.75)
	Not stated	14	(6.57)
**Vaginal sex in the past month**	None	44	(20.66)
	1 time	54	(25.35)
	2 times	44	(20.66)
	3–4 times	44	(20.66)
	5–7 times	12	(5.63)
	≥10 times	4	(1.88)
**Anal sex in the past month**	None	157	(73.71)
	1–23 times	44	(20.66)
	Not stated	12	(5.63)
**Oral sex in the past month**	None	168	(78.87)
	1–8 times	35	(16.43)
	Not stated	10	(4.69)
**Currently on Contraceptive**	Yes	134	(62.91)
	No	64	(30.05)
	Not stated	15	(7.04)
**Method used to prevent pregnancy in last intercourse**	None	49	(23.00)
	Oral contraceptive	13	(6.10)
	Injectables	79	(37.09)
	Other*	57	(26.76)
	Not stated	15	(7.04)
**Ever pregnant**	Yes	47	(22.07)
	No	166	(77.93)
**Experience vaginal discharge**	Yes	138	(64.79)
	No	75	(35.21)
**Experience genital warts, blisters or ulcers**	Yes	43	(20.19)
	No	168	(78.87)
	Not stated	2	(0.94)

*Other includes: Condom = 53; Barrier method/implanon = 3; Condom and barrier method = 1.

### HPV prevalence

**Fig 1:**
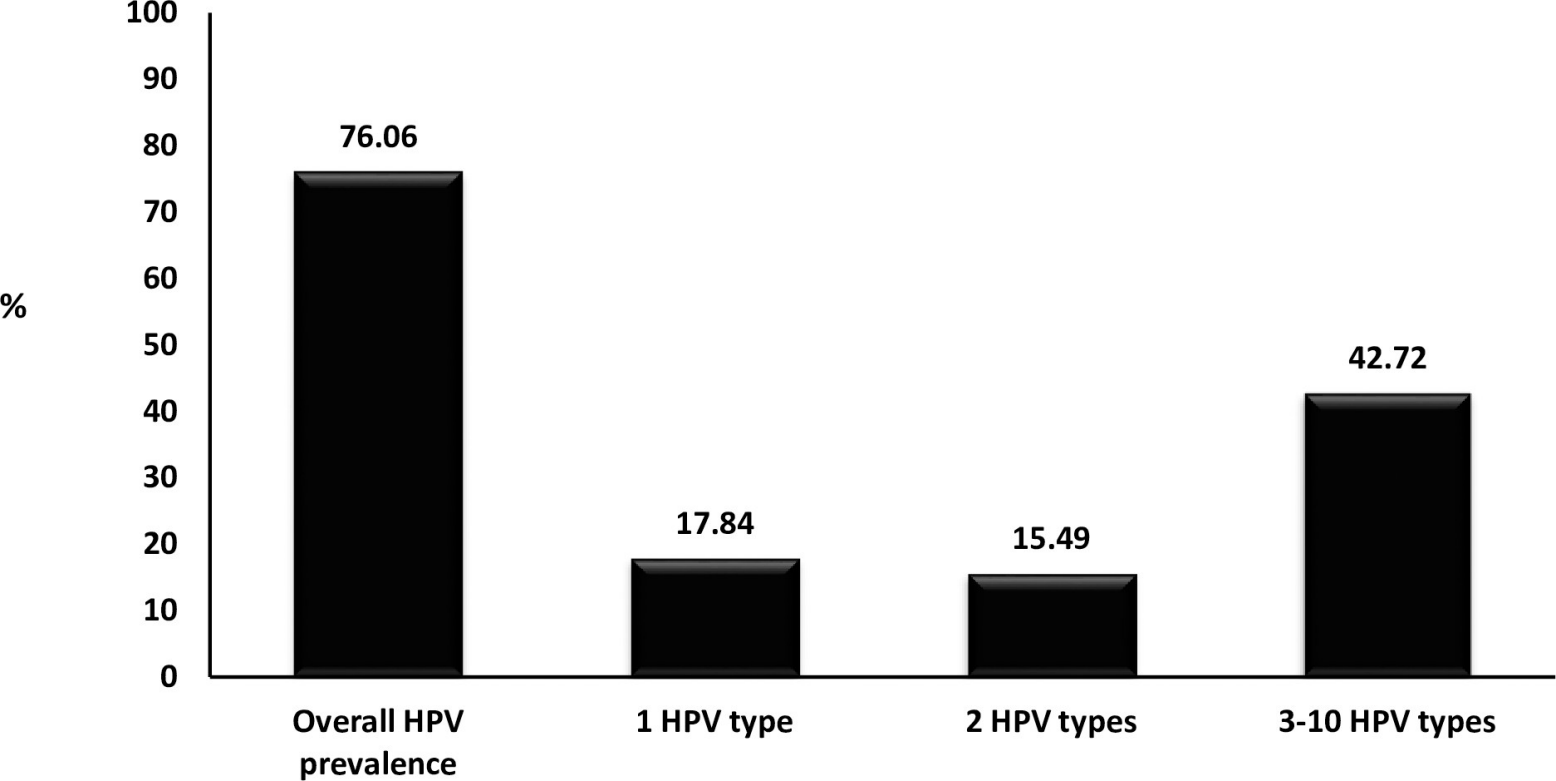
Overall human papillomavirus (HPV) prevalence among sexually active high school female learners of Eastern Cape Province, South Africa.

**Fig 2:**
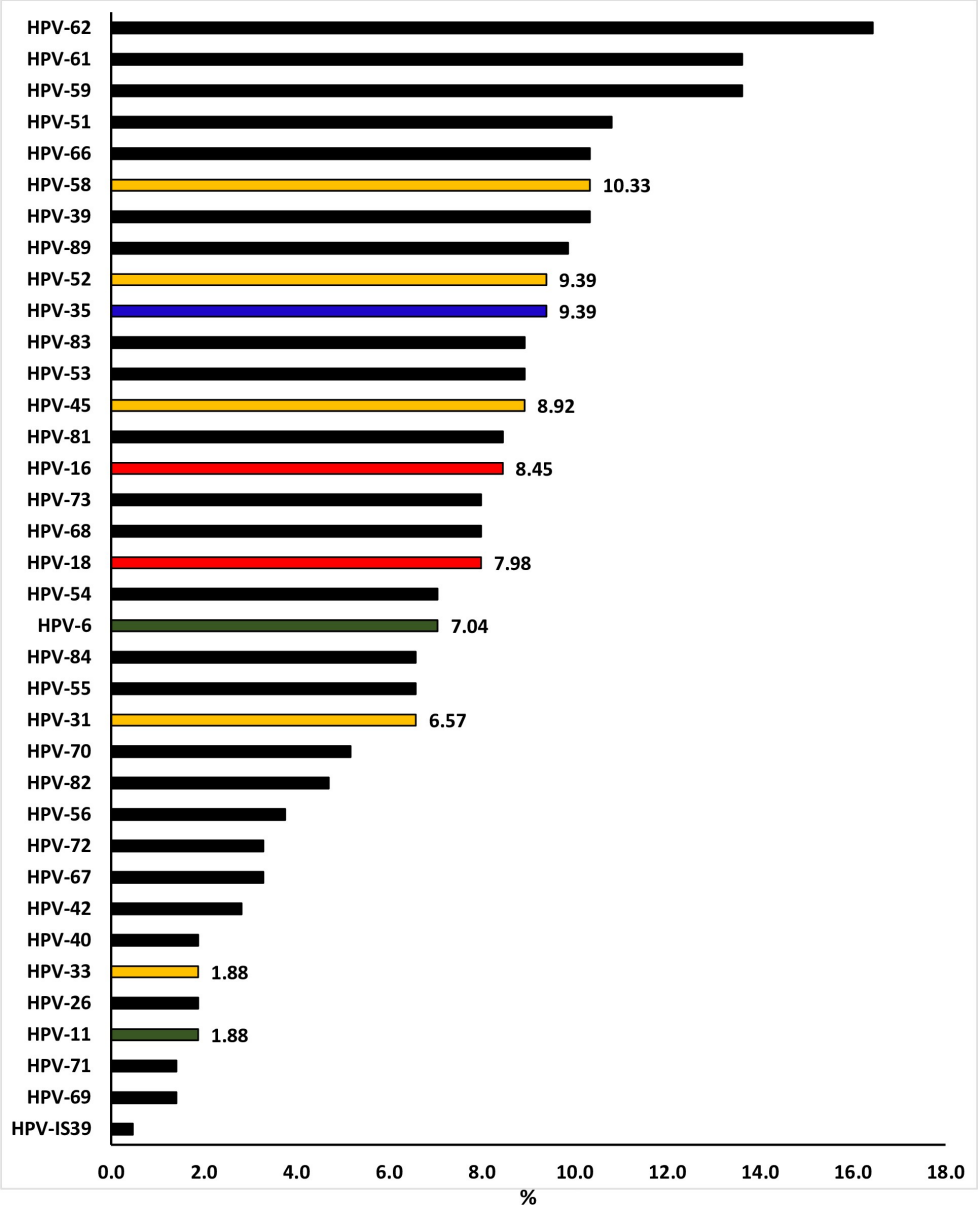
Distribution of human papillomavirus (HPV) genotypes among sexually active high school female learners of Eastern Cape Province, South Africa. In red are HPV types targeted by Cervarix; red-green are HPV types targeted by Gardasil-4; red-green-yellow are HPV types targeted by Gardasil-9. Blue is HPV-35 known to be common in African ancestry women with cervical cancer. Black are types not targeted by any of the current HPV vaccines.

HPV infection was detected in 76.06% (162/213) high school female learners (Table 3). HPV prevalence was found to remain above 70.00% even when stratified according to age groups [15–16 years: 73.68% (14/19); 17–18 years: 76.92% (70/91); 19–20 years: 73.68% (56/76) and 21–25 years: 81.48% (22/27)]. Infection with multiple HPV types (58.22%, 124/213) was more common than single HPV infection (17.84%, 38/213; p<0.0001). Prevalence of HR-HPV types (54.46%, 116/213) was as high as that of LR-HPV types (53.52%, 114/213; p = 0.846). School-Clinic-1 demonstrated higher HPV prevalence (89.86%, 62/69) than School-Clinic-2 (69.44%, 100/144; p = 0.001, Table 3). HPV prevalence was not found to differ between HIV-negative (74.10%, 103/139), and HIV-positive (75.00, 6/8; p = 0.955) or the female learners that were not tested for HIV (80.30%, 53/66; p = 0.332, Table 4). A proportion of 17.84% (38/213) were infected with one HPV type, 15.49% (33/213) were infected with two different HPV types, and 42.72% (91/213) were infected with three to ten different HPV types (Fig 1). The distribution of HPV types detected among high school female learners is presented in Fig 2. The most dominant HPV types were HPV-62 (16.4%), followed by HPV-61 (13.6%), HPV-59 (13.6%), HPV-51 (10.8%, Fig 2).

**Table 3:** Human papillomavirus (HPV) prevalence among sexually active high school female learners, overall and according to study site.

HPV Status	Total, N = 213 n (%; 95%CI)	School-clinic-1, N = 69 n (%; 95%CI)	School-clinic-2, N = 144 n (%; 95%CI)	p-value*
**Any HPV**	162 (76.06; 69.82–81.35)	62 (89.86; 82.73–96.98)	100 (69.44; 61.92–76.97)	**0.001**
**Multiple HPV Infections**	124 (58.22; 51.28–64.92)	46 (66.67; 55.54–77.79)	78 (54.17; 46.03–62.30)	0.083
**Single HPV infection**	38 (17.84; 12.95–23.65)	16 (23.19; 13.23–33.15)	22 (15.28; 9.40–21.15)	0.158
**HR-HPV types**	116 (54.46; 47.52–61.28)	43 (62.32; 50.88–73.75)	73 (50.69; 42.53–58.86)	0.111
**Probable HR-HPV types**	81 (38.03; 31.48–44.91)	31 (44.93; 33.19–56.66)	50 (34.72; 26.95–42.50)	0. 151
**LR-HPV types**	114 (53.52; 46.58–60.36)	42 (60.87; 49.35–72.39)	72 (50.00; 41.83–58.17)	0.137

HR-HPV types: HPV-16, -18, -31, -33, -35, -39, -45, -51, -52, -56, -58 and -59. Probable HR-HPV types: HPV-26, -53, -66, -67, -68, -70, -73 and -82. LR-HPV: HPV-6, -11, -40, 42, -54, -55, -61, -62, -64, -69, -71, -72, -81, -83, -84, -89 (CP6108) and–IS39.

* compares school-clinic-1 and school-clinic-2.

**Table 4:** Factors associated with human papillomavirus (HPV) prevalence among high school female learners of Eastern Cape.

Variable		HPV prevalence	OR (95% CI)	p-value
**Age**		162/213 (76.06)	0.94 (0.78–1.14)	0.540
**Sexual debut age**		162/213 (76.06)	0.96 (0.77–1.19)	0.690
**HIV status**	**Negative**	103/139 (74.10)	ref	
	**Positive**	6/8 (75.00)	1.05 (0.20–5.43	0.955
	**Not tested**	53/66 (80.30)	1.42 (0.70–2.91)	0.332
**Grade**	**12**	47/63 (74.60)	ref	
	**8**	9/12 (75.00)	1.02 (0.25–4.24)	0.977
	**9**	11/17 (64.71)	0.62 (0.20–1.96)	0.420
	**10**	42/55 (76.36)	1.10 (0.47–2.55)	0.825
	**11**	51/64 (79.69)	1.34 (0.58–3.07)	0.496
**Ever consumed alcohol**	**No**	22/38 (57.89)	ref	
	**Yes**	140/175 (80.00)	2.91 (1.38–6.11)	**0.005**
**Number of lifetime sexual partners**	**1**	34/49 (69.39)	ref	
	**≥2**	123/149 (82.55)	2.09 (1.00–4.38)	0.051
**Number of Sexual partners past three months**	**1**	114/141 (80.85)	ref	
	**≥2**	23/25 (92.00)	2.72 (0.60–12.26)	0.192
**Ever pregnant**	**No**	126/166 (75.90)	ref	
	**Yes**	36/47 (76.70)	1.04 (0.48–2.23)	0.922
**Vaginal discharge/itching**	**No**	54/75 (72.00)	ref	
	**Yes**	108/138 (78.26)	1.40 (0.73–2.67)	0.308
**Genital Warts/Blisters**	**Yes**	31/43 (72.09)	ref	
	**No**	129/168 (76.79)	1.28 (0.60–2.73)	0.522
**Circumcised Boyfriend**	**No**	41/56 (73.21)	ref	
	**Yes**	120/153 (78.43)	1.33 (0.66–2.69)	0.428
**Current contraceptive**	**None**	39/49 (79.59)	ref	
	**Oral contraceptives**	11/13 (84.62)	1.41 (0.27–7.41)	0.757
	**Injectable contraceptives**	64/79 (81.01)	1.09 (0.45–2.67)	0.844
	**Other**	43/57 (75.44)	0.79 (0.31–1.98)	0.611

### HPV type distribution and prevalence of HPV types targeted by HPV vaccines

**Fig 3:**
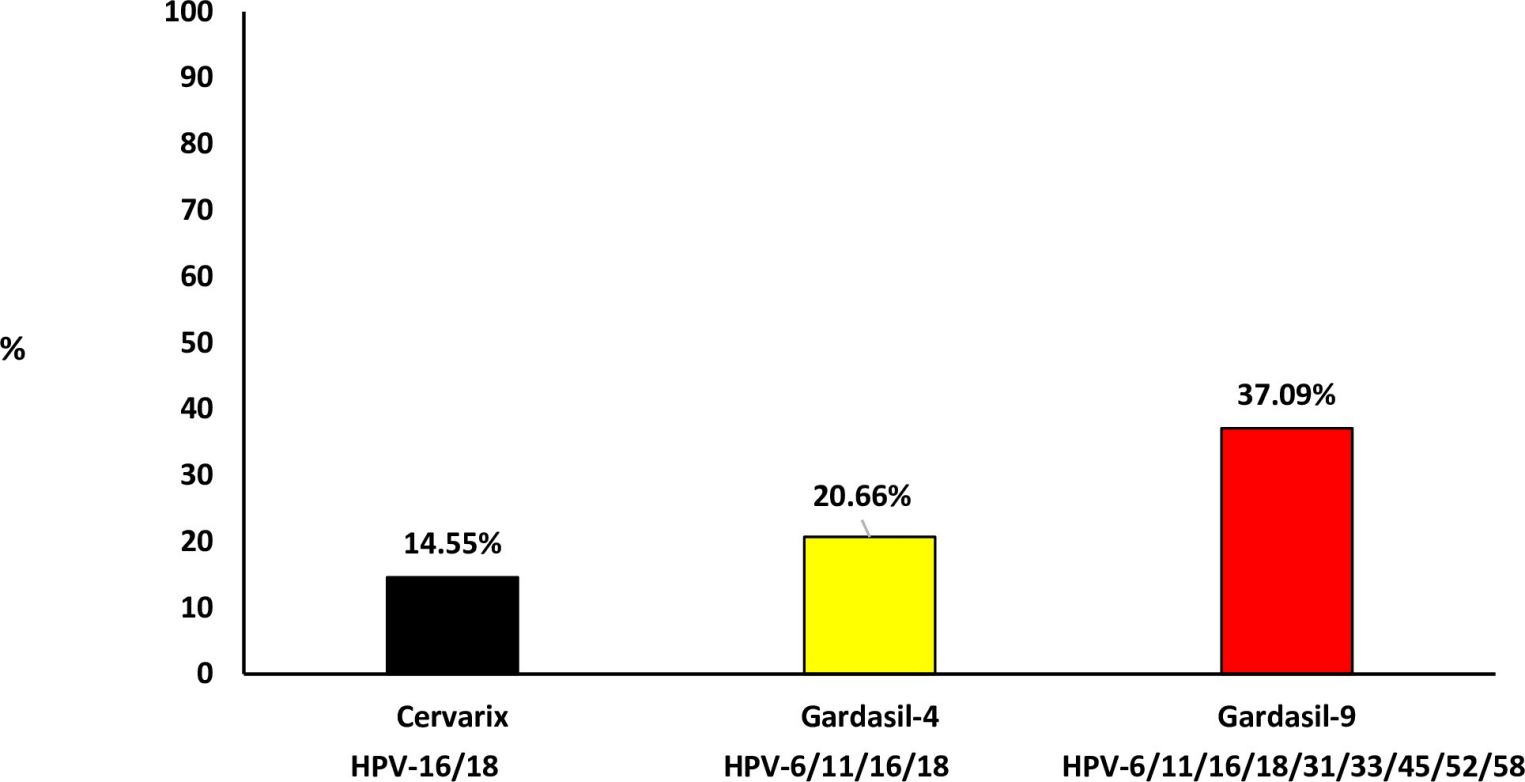
Prevalence of Human papillomavirus (HPV) types targeted by current commercial HPV vaccines among sexually active high school female learners. (Cervarix vaccine targets HPV-16/18; Gardasil-4 vaccine targets HPV-6/11/16/18 and Gardasil-9 vaccine targets HPV-6/11/16/18/31/33/45/52/58).

In Fig 2, the prevalence of individual HPV types targeted by current commercial HPV vaccines are indicated. Among high school female learners, HPV-58 (10.33%) was the most commonly detected type targeted by the current commercial HPV vaccine, followed by HPV-52 (9.39%), HPV-45 (8.92%), HPV-16 (8.45%), HPV-18 (7.98%), HPV-6 (7.04%), HPV-31 (6.57%), HPV-33 (1.88%), and HPV-11 (1.88%, Fig 2). HPV type(s) targeted by the Cervarix® HPV vaccine (HPV-16 and/or 18), currently used in the South African school-based HPV vaccination program, were detected in 14.55% (31/213), those targeted by Gardasil®4 (HPV-6, -11, -16 and/or -18) were detected in 20.66% (44/213), and those targeted by Gardasil®9 (HPV-6, -11, -16, -18, -31, -33, -45, -52 and/or -58) were detected in 37.09% (79/213, Fig 3). Among the types not targeted by current commercial HPV vaccines but commonly detected in cervical cancer cases among women of African ancestry origin, HPV-35 was detected in 9.39% female learners (Fig 2).

### Factors associated with HPV prevalence among high school female learners

Female learners who reported having ever consumed alcohol had a significantly higher risk of HPV infection (OR: 2.91, 95% CI: 1.38–6.11, p = 0.005). Participants who reported ≥2 number of lifetime sexual partners (OR: 2.09, 95% CI: 1.00–4.38, p = 0.051) sexual partners past three months (OR: 2.72, 95% CI: 0.60–12.26, p = 0.192) had a higher risk of HPV infection but not statistically significant. Age, sexual debut age, and school grade were not found to be associated with HPV infection among female learners (Table 4).

## Discussion

According to our knowledge, this is the first peer-reviewed report on HPV prevalence and genotype distribution among female learners attending high schools in the rural areas of Eastern Cape Province of South Africa. High overall HPV prevalence (76.06%) and infection with 3–10 different HPV types (42.72%) are of public concern in this population. Among those that tested for HIV infection, the HPV prevalence was still found to be high among the HIV-negative group (74.10%). Giuliano et al. report similar HPV prevalence (71.4%) among HIV-negative women between the age of 16 and 24 years residing in the Western Cape Province of South Africa. The 94% of the Giuliano et al. study participants were of black ethnicity [17]. The observed HPV prevalence was higher than the one previously reported among HIV-negative adolescents and young adults in the Western Cape (68.2%) and Gauteng (65.0%) provinces of South Africa [19]. However, it was in the range of the HPV prevalence previously reported in literature among South African adolescents and young women [14–20]. Compared with the population outside South Africa, the observed HPV prevalence was lower (60.3%) among HPV unvaccinated Colombian women between the ages of 18–25 years. The detection of multiple HPV infections was also common than a single HPV infection. It is important to note that the Roche Linear Array HPV Genotyping Test was also used in a Colombian study [39].

Girls who were vaccinated in 2014 when South Africa initially introduced the HPV school-based vaccination program were 14 years of age in 2019 when this current study was conducted. Therefore, the probability that some of the study participants would have received the HPV vaccination a few years before is low as the current study participants were between the age of 15 and 22 years. None of the study participants reported having received HPV vaccination at primary school. The high prevalence of HPV types targeted by Cervarix® HPV vaccine (HPV-16 and/or 18; 14.55%) and Gardasil®9 (HPV-6, -11, -16, -18, -31, -33, -45, -52 and/or -58; 37.09%) was similar to the one previously reported among HIV-negative Western Cape (Cape Town) and Gauteng (Soweto) adolescents and young women (18.6% and 38.5% respectively) [19]. The high prevalence of HPV types targeted by Gardasil®9 promotes the introduction of Gardasil®9 HPV vaccine as it will offer protection to more HPV types associated with cancers [22]. HPV-35, known to be common in African ancestry women with cervical cancer [33,34], was common (9.4%) in this study.

It is not clear why the overall HPV prevalence was found to differ between school-clinic-1 and school-clinic-2 as the participants’ demographic data was not found to differ; both the school-clinic sites are located in rural areas far from town. Participants who reported having ever consumed alcohol had a higher risk of HPV infection, which could indicate high-risk sexual behaviour [40]. However, few participants reported being drunk during the last sexual intercourse. Only 38.19% of learners reported having used condoms during their last sexual encounter, and 32.32% were not using any contraceptives. The South African National Youth Risk Behaviour Survey (SANYRBS) 2011 among learners attending high schools in Eastern Cape Province, reported that 37.1% had had sex in their lifetime, 43.0% had at least 2 sexual partners in their lifetime, and 28.7% mostly used condoms during sexual intercourse [41]. The low condom usage among learners in the SANYRBS and the current study suggest that learners are highly exposed to sexually transmitted diseases (STDs) and adolescent pregnancy. The observed high burden of HPV infection among female high school learners emphasizes the importance and need for HPV awareness programs in schools and communities. In view of the majority of learners reported to be sexually active, knowledge about different STDs, risk factors of acquiring STDs, and disease associated with sex is a fundamental need for this population [42]. HPV awareness programs may also support vaccination and cervical cancer screening programs.

It is acknowledged that the study population was from two communities and does not represent the population of Eastern Cape Province, and cannot be generalised. Despite these limitations, the information reported remains essential for this province and South Africa as there is currently limited HPV information on this population. There are few chances that the participants could have received the HPV vaccine during the national school HPV vaccination program or elsewhere. However, the possibility of receiving the HPV vaccine is not completely ruled out because the vaccines were available in the country.

## Conclusion

High HPV prevalence was observed among high school female learners. The high prevalence of HPV types targeted by the Gardasil®9 vaccine encourages the introduction of the Gardasil®9 vaccine. It is envisaged that these results will contribute to HPV baseline data among adolescents and young women of Eastern Cape Province and will be utilized to evaluate the impact of HPV vaccination. Research or surveillance projects to monitor HPV prevalence and distribution among HPV vaccinated and unvaccinated adolescents and young women are necessary to monitor the impact of HPV vaccination in South Africa.

## Supporting information

S1 Data (XLSX)
